# COMP–PMEPA1 axis promotes epithelial‐to‐mesenchymal transition in breast cancer cells

**DOI:** 10.1002/1878-0261.70221

**Published:** 2026-02-13

**Authors:** Konstantinos S. Papadakos, Gilar Gorji‐bahri, Lejla Gradjan, Julia Zajac, Kacper Gil, Yvonne Thokozile Nduku, Anna M. Blom

**Affiliations:** ^1^ Division of Medical Protein Chemistry, Department of Translational Medicine Lund University Malmö Sweden

**Keywords:** breast cancer, COMP, EMT, metastasis, PMEPA1

## Abstract

Epithelial‐to‐mesenchymal transition (EMT), driven by cues from the tumor microenvironment, is a critical initiator of metastatic progression. In breast cancer patients, elevated expression of cartilage oligomeric matrix protein (COMP) is associated with shorter survival and increased metastatic risk. Here, we investigated the role of COMP in regulating EMT in breast cancer. Breast cancer cells treated with recombinant COMP or engineered to overexpress *COMP* exhibited a marked decrease in the epithelial marker *CDH1* and an increase in mesenchymal markers such as *VIM* and *VCAN*. Consistent with these *in vitro* findings, *COMP*‐expressing xenograft tumor tissues showed enhanced EMT characteristics. Functionally, COMP promoted increased migration and invasion of breast cancer cells in both autocrine and paracrine manners, dependent on its thrombospondin (TSP) and C‐terminal domains. We further identified protein TMEPAI (encoded by gene *PMEPA1*) as a functional COMP‐binding partner that mediates COMP‐induced EMT, primarily through interaction with the TSP domain of COMP. Mechanistically, COMP shifted transforming growth factor beta (TGFβ) signaling from canonical phosphorylated mothers against decapentaplegic homolog 2/3 (pSMAD2/3) activation toward pSMAD1/5, likely through its interaction with PMEPA1. This study suggests the COMP–PMEPA1 axis as a new driver of EMT in breast cancer models.

## Graphical abstract

This study reveals that cartilage oligomeric matrix protein (COMP) promotes epithelial‐to‐mesenchymal transition (EMT) in breast cancer. We identify PMEPA1 (protein TMEPAI) as a novel COMP‐binding partner that mediates EMT via binding to the TSP domains of COMP, establishing the COMP–PMEPA1 axis as a key EMT driver in breast cancer.

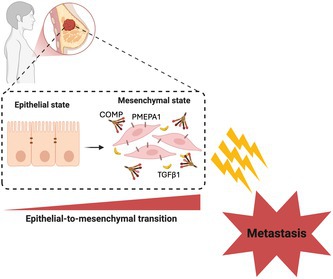

**Abbreviations**
BCA: bicinchoninic acid assay
BSA: bovine serum albumin
CFSE: carboxyfluorescein succinimidyl ester
COMP: cartilage oligomeric matrix protein
EMT: epithelial‐to‐mesenchymal transition
ER: endoplasmic reticulum
ER^+^/PR^+^
: estrogen receptor/progesterone receptor‐positive
PET: polyethylene terephthalate
PLA: proximity ligation assay
PMEPA1: prostate transmembrane protein, androgen induced 1
RIPA: radioimmunoprecipitation assay
RT‐qPCR: reverse transcription‐quantitative real‐time polymerase chain reaction
TNBC: triple‐ negative breast cancer
TNM: tumor, node, metastasis
TSP: thrombospondin

## 1 Introduction

Despite all the efforts to reduce cancer burden, breast cancer remains the leading cause of death in women, accounting for 15% of cancer‐related mortality cases and 24% of cases globally [1]. The most common breast cancer metastatic sites include bone, liver, lungs, and brain [2]. Metastasis is the leading cause of cancer‐related death and involves a complex, multistep process. It begins with local invasion, driven primarily by the epithelial‐to‐mesenchymal transition (EMT), which enables cancer cells to penetrate surrounding tissue. In the second stage, intravasation, cancer cells enter the blood or lymphatic circulation. Once in circulation, they face a hostile environment, including shear stress and immune attack. Only a few survive to extravasate, where they exit the vessels and form micrometastases in distant tissues. These may remain dormant before progressing to the final stage, colonization, where cancer cells adapt to the new microenvironment and form vascularized secondary tumors [3].

EMT is a reversible dynamic differentiation process in which epithelial cells lose polarity and cell adhesion to acquire a mesenchymal, fibroblast‐like phenotype, characterized by increased motility and invasiveness. However, it is not a simple binary phenomenon of cells transitioning between epithelial and mesenchymal states. Growing evidence indicates an intermediate phase of hybrid or partial EMT where cells co‐express epithelial and mesenchymal markers and display an enhanced metastatic capacity and stemness compared with cells in a fully mesenchymal state [4, 5, 6]. EMT is essential in embryogenesis and wound healing, but also plays a key role in fibrosis and cancer metastasis. It is triggered by microenvironmental cues such as TGFβ, tissue stiffness, and hypoxia, leading to widespread changes in gene expression and cell behavior [7, 8, 9]. The process can be reversed via mesenchymal to epithelial transition, for instance, in metastatic colonization [10]. Various signaling pathways induce EMT in breast cancer models *in vitro* and *in vivo*, such as hypoxia‐mediated Notch‐Slug and Snail axis [11], the involvement of cytosolic calcium concentration in EGF and hypoxia‐induced EMT [12], Six1‐Hh/Gli axis‐mediated EMT induction in a paracrine manner in non‐EMT cells [13], IL6 secretion by adipocytes [14], and TGFβ secretion by cancer‐associated fibroblasts [15].

Cartilage oligomeric matrix protein (COMP) is a pentameric glycoprotein that plays a pivotal role in the extracellular matrix organization of cartilage and its stiffness. Following our initial unexpected observation regarding COMP expression in breast cancer [16], COMP expression was detected in tumor tissues of patients with prostate cancer [17], pancreatobiliary adenocarcinoma [18], colon cancer [19, 20], ovarian cancer [21], esophageal and gastric adenocarcinoma [22], and hepatocellular carcinoma [23]. In breast cancer patients, COMP expression by cancer cells was an independent prognostic factor of patients' recurrence‐free survival. A xenograft mouse model, in which COMP‐expressing MDA‐MB‐231 cells were orthotopically injected into SCID mice, developed significantly larger primary tumors and detectable metastases to the lungs and lymph nodes compared to control (mock) mice. *In vitro*, COMP‐expressing breast cancer cells exhibited a higher invasion ability through the extracellular matrix, which was partly facilitated by the increased expression of matrix metalloproteinase 9 (MMP9) [16].

Prostate transmembrane protein androgen induced 1 (gene: *PMEPA1*, protein: protein TMEPAI) was first identified in prostate cancer, where it is predominantly expressed [24]. *PMEPA1* expression is regulated by the androgen receptor and associated with DNA methylation and TGFβ signaling pathway [25, 26]. Androgen receptor‐positive prostate cancer cell lines exhibit higher *PMEPA1* methylation and reduced *PMEPA1* expression. Further, methyl transferase inhibitor restores PMEPA1 expression, emphasizing the regulatory role of DNA methylation in PMEPA1 expression [25]. Survival analysis of breast cancer patient cohorts revealed a positive correlation between lower *PMEPA1* expression and shorter recurrence‐free survival. In addition, *PMEPA1* expression was increased by TGFβ in prostate cancer cells, while it negatively regulates the TGFβ signaling pathway [26]. In contrast, high expression of *PMEPA1* in colorectal cancer was correlated with shorter overall survival and TNM stages [27]. These controversies indicate the context‐dependent effect of *PMEPA1* expression on cancer progression and the necessity of further investigation.

In this study, we aimed to understand how COMP expression in primary tumors contributes to a metastatic advantage through local tissue invasion and increased invasiveness. Considering the important contribution of EMT to metastasis, we focused on the EMT pathway and found that COMP‐expressing breast cancer cells exhibited molecular characteristics consistent with EMT. Our results show that PMEPA1 is overexpressed in COMP‐expressing breast cancer cells, interacts with COMP, primarily via its thrombospondin (TSP) domains, and contributes to COMP‐driven invasiveness and EMT‐related molecular characteristics. Mechanistically, COMP was found to change the activity of the canonical TGFβ signaling pathway, likely via interaction with PMEPA1.

## 2 Materials and methods

### 2.1 Cell lines and proteins

Triple‐negative breast cancer (TNBC) cell lines, BT‐20 (CVCL_0178), MDA‐MB‐231 (CVCL_0062), and HS‐578 T (CVCL_0332) were purchased from Cytion, ATCC, and DSMZ, respectively. These three cell lines were cultured in DMEM high glucose (Cytiva, Logan, UT, USA) supplemented with 10% fetal bovine serum (FBS; ATCC), and 1% penicillin (100 U·mL^−1^) and streptomycin (100 μg·mL^−1^) (Cytiva). MCF7 cell line (CVCL_0031), an ER^+^/PR^+^ breast cancer cell line, was purchased from ATCC and cultured in DMEM low glucose (Cytiva) supplemented with MEM non‐essentials amino acids (Gibco, Paisley, UK), human recombinant insulin (0.01 mg·mL^−1^) (Gibco, Grand Island, NY, USA), 10% FBS, and 1% penicillin (100 U·mL^−1^) and streptomycin (100 μg·mL^−1^). Cells expressing COMP and the mock controls were generated by transfection of COMP‐cloned pcDNA3 vector or an empty vector and were cultured in the presence of 0.7 mg·mL^−1^ geneticin (G418) (Gibco). All cell lines were directly purchased from the mentioned vendors and were authenticated with short tandem repeat (STR) profiling according to the ANSI Standard established by the ATCC Standards Development Organization. Cells were used for the experiments at passage numbers below ten. All cells in culture were routinely tested for mycoplasma contamination by Eurofins Genomics and confirmed to be mycoplasma‐free. The recombinant COMP (r‐COMP) was expressed in eukaryotic FreeStyle 293‐F cells (Gibco) with a His‐tag and purified by affinity chromatography as described before [21].

### 2.2 RT‐qPCR and EMT qPCR array

Gene expression was investigated by RT‐qPCR, as described before [21]. Total RNA extraction was performed using the RNeasy Plus Mini Kit (Qiagen, Hilden, Germany) according to the manufacturer's instructions. cDNA synthesis was carried out using Superscript IV reverse transcriptase (Invitrogen, Vilnius, Lithuania) in combination with oligo (dT)18 primer (Thermo Scientific, Vilnius, Lithuania), dNTP mix (Thermo Scientific), 5× SSIV buffer, and RNaseOUT ribonuclease inhibitor (Invitrogen, Carlsbad, CA, USA). For the RT‐qPCR experiment, TaqMan gene expression master mix (Applied Biosystems, Vilnius, Lithuania) with primers and probes, listed in Table S1, was used. The amplification was carried out using the Viia 7 real‐time PCR system (Applied Biosystems). The gene expression was calculated by the 2^−(ΔCt)^ method.

For EMT qPCR array, BT‐20 cells were seeded in a 6‐well plate to achieve 70% confluency the following day. Total RNA was isolated using RNeasy Plus Mini Kit (Qiagen) according to the manufacturer's instructions. cDNA synthesis was performed using the iScript (Bio‐Rad, Hercules, CA, USA) reverse transcription kit according to the manufacturer's instructions. The qPCR array was conducted using the EMT PrimePCR Custom Plate, 10 034 487, 384‐well (Bio‐Rad), and the SsoAdvanced Universal SYBR Green Supermix (Bio‐Rad), following the manufacturer's instructions. The amplification was carried out using the Viia 7 real‐time PCR system (Applied Biosystems), following the PrimePCR cycling protocol. The gene expression was calculated by the 2^−(ΔCt)^ method.

### 2.3 Protein analyses

Cells were seeded in 6‐well plates to reach 70–80% confluency on the day of lysate collection. For western blot analysis, cells were lysed by radioimmunoprecipitation assay buffer (RIPA buffer) (25 mm Tris/HCl pH 7.6, 150 mm NaCl, 1% Triton X‐100, 1% Sodium‐deoxycholate, 0.1% SDS) supplemented with Halt protease and phosphatase inhibitors (Pierce, Rockford, IL, USA). Protein concentration was quantified using the BCA assay kit (Pierce), and equal amounts of total protein were loaded onto mini‐protean TGX stain‐free precast gels (Bio‐Rad). Separated proteins were transferred to PVDF membranes utilizing the Trans‐blot transfer system (Bio‐Rad). The PVDF membranes were incubated overnight with the appropriate primary antibody (Table S2), then washed and incubated in the corresponding secondary antibody for 1 h at room temperature (Table S2). Membranes were then developed with the Immobilon Western chemiluminescent HRP substrate (Millipore, Burlington, MA, USA), and photos were captured using the ChemiDoc Imaging System (Bio‐Rad).

For co‐immunoprecipitation, cells were lysed with ice‐cold NP40 lysis buffer (150 mm NaCl, 50 mm Tris/HCl pH = 7.5, 1% NP40) and equal amounts of protein were incubated overnight with anti‐PMEPA1 antibody. The next day, 50 μL of Dynabeads protein G (Invitrogen) was added to the lysates and incubated for 2 h. Beads were washed three times with ice‐cold NP40 lysis buffer, and proteins were finally extracted using Laemmli buffer (0.5 M Tris/HCl, 1 g·L^−1^ bromophenol blue, 33% glycerol). COMP protein was detected by western blot analysis.

For sandwich ELISA, the Nunc Maxisorp plates (Thermo Fisher Scientific, Roskilde, Denmark) were coated with 100 μL of 5 μg·mL^−1^ mouse anti‐PMEPA1 antibody diluted in PBS overnight at 4 °C. The next day, cells were lysed with ice‐cold NP40 lysis buffer, and equal amounts of protein lysate were loaded in the wells, which had previously been blocked with 3% bovine serum albumin (BSA) (AppliChem, Darmstadt, Germany) for 1 h at room temperature. Plates were incubated overnight with gentle agitation at 4 °C. The day after, plates were washed (50 mm Tris/HCl, 150 nm NaCl, 0.05% Tween 20) and rabbit polyclonal antibody against COMP was added to the wells for 1 h at room temperature. The secondary anti‐rabbit antibody was added to the wells for 1 h at room temperature. Plates were finally washed and incubated with TMB substrate (Kementec, Taastrup, Denmark), and the absorbance was measured at 450 nm using the Cytation 5 (BioTek, Winooski, VT, USA).

### 2.4 Proximity ligation assay (PLA)

The PLA was performed using the Duolink kit (Sigma, St.Louis, MO, USA) according to the manufacturer's instructions. Cells were seeded in a 12‐well chamber slide (Ibidi) to reach 50% confluency the next day. Cells were washed with PBS, then fixed with 4% PFA (Merck, Darmstadt, Germany) in PBS for 5 min, and washed with PBS, and the PFA was then neutralized with 50 mm NH_4_Cl. Subsequently, the cell membrane was permeabilized using 0.1% Triton X‐100 for 5 min. Cells were then blocked with 3% BSA (AppliChem, Darmstadt, Germany) in PBS for 15 min at room temperature. The cells were incubated with appropriate primary antibodies (Table S2) diluted in PBS containing 3% BSA (AppliChem) and incubated overnight at 4 °C. The next day, cells were washed with PBS containing 0.1% Triton X‐100, and the protein interactions were visualized using the Duolink kit (Sigma), following the manufacturer's instructions. The z‐stack images were captured with the LSM 800 with Airyscan (Zeiss, Göttingen, Germany) confocal microscope. The quantification of the dots per cell was calculated with the imagej software.

### 2.5 Tumor staining

Archived tissues from a xenograft mouse model [16] were used for the immunofluorescence staining or the PLA assay. Paraffin‐embedded primary tumors were cut and transferred to Superfrost Plus Adhesion Microscope slides (Epredia, Breda, Netherlands). Tissues were rehydrated by serial incubation in Xylene for 10 min twice, absolute ethanol for 5 min twice, 95% ethanol for 5 min twice, 70% ethanol for 5 min, water for 5 min twice, and PBS for 3 min three times. Antigen retrieval was performed in citrate buffer (10 mm sodium citrate, 0.05% Tween 20, pH = 6.0) at 90 °C for 10 min using a cooker pressure. Tissues were permeabilized using 5% Triton X‐100 and blocked using 5% normal donkey serum (Jackson ImmunoResearch) in PBS for 2 h at 4 °C. Tissues were then incubated with the appropriate primary antibodies (Table S2) overnight at 4 °C. For immunofluorescence staining, tissues were incubated with the appropriate secondary antibodies for 4 h at 4 °C (Table S2) and DAPI mounting medium (Invitrogen).

For PLA assay, tissues were washed with PBS containing 0.1% Triton X‐100, and the protein interactions were visualized utilizing the Duolink kit (Sigma), following the manufacturer's instructions. Finally, tissues were mounted using the mounting medium (Sigma). The images were captured with the LSM 800 with Airyscan (Zeiss) confocal microscope. Image analysis was performed using imagej software.

### 2.6 *PMEPA1* downregulation using siRNA

Breast cancer cells were seeded in a 6‐well plate to reach a 70% confluency the following day. On the day of transfection, the medium was replaced with a fresh 1.6 mL of complete culture medium. The transfection complex was formed in 400 μL of Opti‐MEM medium (Gibco) using 16 μL of Lipofectamine RNAiMAX (Invitrogen) and the appropriate concentration of the siRNA against *PMEPA1* ON‐TARGETplus Human *PMEPA1* pool (Dharmacon) or the negative control ON‐TARGETplus non‐targeting pool, control 1 (Dharmacon). After optimization, 80 nm and 60 nm of siRNAs were used for the BT‐20 cells and HS‐578 T cells, respectively. Downregulation of *PMEPA1* was confirmed at the mRNA level by RT‐qPCR and protein level for the encoded PMEPA1 by western blot analysis.

### 2.7 Migration and invasion assays

Migration and invasion assays were performed as described [21]. Cell culture PET inserts with 8 μm pores (Falcon, Durham, NC, USA), together with the companion 24‐well plate (Falcon), were used for the migration assay. The invasion assay was performed using the BioCoat Matrigel invasion chamber (Corning, Bedford, MA, USA) with 8 μm pores. In all cases, 700 μL medium containing 10% FBS was added to each well of the 24‐well plate. For the migration or the invasion assay, 8 × 10^4^ BT‐20 cells and 4 × 10^4^ HS‐578 T, diluted in medium containing 1% FBS were seeded in the inserts. After 48 h, the non‐migrated cells were removed by cotton‐tipped applicators. The migrated cells were then fixed with 4% PFA (Merck) in PBS and stained with 0.5% crystal violet (Merck). Images were captured using the EVOS inverted microscope, and the total number of migrated cells was calculated using the qupath software [28].

For siRNA experiments, 8 × 10^5^ cells were seeded in a 6‐well plate for 24 h. The next day, cells were transfected with the siRNA against *PMEPA1* or the scrambled control, as described above. After 24 h, cells were detached using trypsin (Cytiva), centrifuged for 5 min at 300 ***g***, and the appropriate number of cells was then seeded in the inserts as described above.

For the cell labelling with CFSE, mock cells were incubated with 5 μm CFSE (Invitrogen) for 20 min at room temperature, according to the manufacturer's instructions. The labeled cells were mixed with the COMP‐expressing cells at varying proportions, ranging from 10 to 50%. Control samples with only mock CFSE‐labeled cells were also included. Images were captured using the fluorescent microscope (Zeiss, AX10), and the total number of migrated cells was calculated using the qupath software. To perform the statistical analysis for the CFSE‐labeled coculture assay, the number of migrated CFSE‐labeled mock cells at each cell ratio was initially divided by the total number of seeded cells, and they were subsequently normalized to the control samples.

### 2.8 Wound healing assay

Cells were seeded in a 6‐well plate to achieve a 90% confluent monolayer by the day of the experiment. The medium was exchanged with CO_2_‐independent Medium (Gibco), supplemented with 10% FBS (ATCC), 2 nm L‐Glutamine (Cytiva) and 1% penicillin (100 U·mL^−1^) and streptomycin (100 μg·mL^−1^) (Cytiva). A full‐depth straight‐line scratch was introduced in the middle of the confluent cell layer using a pipette tip. Images were captured every 30 min for 20 h using the Cytation 5 (BioTek). The Cytation 5 system was configured to center images on a predefined beacon in each well. At each time point, the beacon was re‐identified, and focus and image brightness were automatically adjusted. Consequently, unprocessed images may vary in brightness between wells and over time, including due to changes in culture medium color. imagej software was utilized to measure the area of the wound in each image. The wound closure percentage was calculated as follows: Wound closure (%) = [(Wound area at 0 h−wound area at 10 h or 20 h)/wound area 0 h] × 100. To perform the wound healing assay with siRNA‐transfected cells, cells were seeded in a 6‐well plate to reach 70% confluency in 24 h. Cells were then transfected with the siRNA against *PMEPA1*, or the scrambled control, and the assay was performed as described above.

### 2.9 Ethics approval

No animals were directly used in the current study. Only archived tissues from a previous study described in detail in reference [16] were used. The animal experiments described in reference [16] were approved by the local Ethical Committees for Animal Experimentation in Lund, application (4349/2020).

### 2.10 Databases analyses

For co‐expression analysis, mRNA expression of breast cancer patients (*n* = 1084) derived from the TCGA, PanCancer Atlas was analyzed via the cBioportal platform [29]. For survival analysis with *COMP* and *PMEPA1* co‐expression, breast cancer patients were stratified by median expression of *COMP* and *PMEPA1* and grouped into low *COMP* and *PMEPA1*, and high *COMP* and *PMEPA1* co‐expressing groups, using the GEPIA2 online tool [30], which covers RNA sequencing data from the TCGA and GTEx projects. To evaluate the dependency of prognostic effect on individual *COMP* or *PMEPA1* expression, samples were initially stratified by *COMP* low and *COMP* high expression based on the median expression through the cBioportal platform using the TCGA, PanCancer Atlas; mRNA expression. Then, in each group, samples were divided into *PMEPA1* low and *PMEPA1* high expression by median, and overall survival and progression‐free survival were analyzed. Same survival analyses were performed in *PMEPA1* low and *PMEPA1* high groups with *COMP* expression as the second factor.

### 2.11 Statistical analyses

Statistical analyses and graph generation were performed using graphpad prism version 10. *T*‐tests were used for comparisons between two groups. For comparisons involving more than two groups, one‐way ANOVA was used for single‐variable conditions, and two‐way ANOVA was used for experiments with more than one variable.

## 3 Results

### 3.1 COMP induces EMT marker expression and promotes a mesenchymal phenotype

**Fig. 1:**
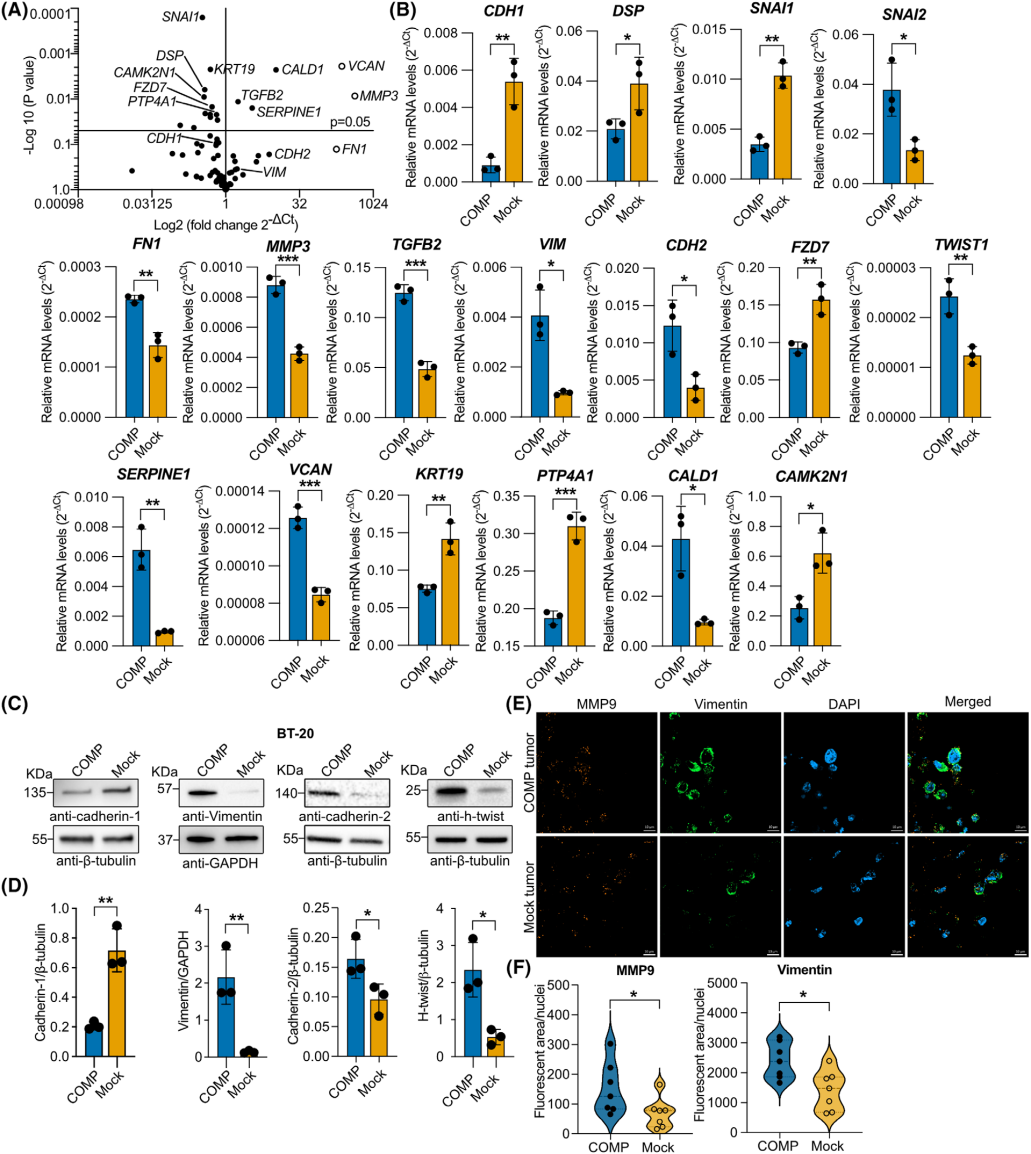
COMP expression by breast cancer cells induced the expression of EMT‐related markers in favor of mesenchymal status. (A) EMT PrimePCR array in COMP‐BT20 cells compared with the mock control cells. Volcano plot was used to represent the data from three independent experiments (*n* = 3). *GAPDH* was used as a reference gene, and white dots represent pseudo‐values, as they were expressed only in COMP‐BT20 cells. Multiple unpaired t‐tests were used for statistical analyses. (B–D) The expression analyses of EMT markers by RT‐qPCR at mRNA level in COMP‐BT20 cells versus the mock control using *HPRT* as a reference gene (B), or by western blot at the protein level (C, D). *P*‐values were calculated using a *t*‐test. Data represent mean ± standard deviation from three independent experiments (*n* = 3). (E) An immunofluorescence staining for two EMT markers, MMP9 and Vimentin in paraffin‐embedded tumor tissues collected from a previously performed xenograft mouse model [16] (Scale bar: 10 μm), with quantification in (F). Each symbol in the graph represents a tumor from an individual mouse (*n* = 7). The Mann–Whitney test was used for the statistical analysis. *P ≤ 0.05, **P ≤ 0.01, and ***P ≤ 0.001. EMT: Epithelial‐to‐mesenchymal transition.

The EMT International Association (TEMTIA) recommends combining phenotypic and molecular markers to experimentally define EMT [31]. Among 87 genes examined by EMT‐specific marker qPCR array, the expression of 17 genes was significantly affected by COMP expression in BT‐20 cells (COMP‐BT20), with 12 genes downregulated and 5 genes upregulated compared with the mock cells (Fig. 1A). The expression of 17 selected genes was further examined in these cells (Fig. 1B). The expression of mesenchymal markers, including Vimentin (*VIM*), cadherin 2 (*CDH2*), snail family transcriptional repressor 2 (*SNAI2*), fibronectin (*FN1*), matrix metallopeptidase 3 (*MMP3*), transforming growth factor beta 2 (*TGFΒ2*), twist family bHLH transcription factor 1 (*TWIST1*), versican (*VCAN*), serpin family E member 1 (*SERPINE1*), and caldesmon 1 (*CALD1*) was significantly upregulated in COMP‐BT20 cells compared with the mock control. Simultaneously, several epithelial markers, including cadherin 1 (*CDH1*), desmoplakin (*DSP*), and keratin 19 (*KRT19*), were significantly downregulated. In addition, frizzled class receptor 7 (*FZD7*), snail family transcriptional repressor 1 (*SNAI1*), protein tyrosine phosphatase 4A1 (*PTP4A1*), Calcium/calmodulin dependent protein kinase II inhibitor 1 (*CAMK2N1*), that is, genes commonly associated with the mesenchymal phenotype, were significantly downregulated in COMP‐BT20 cells compared with the mock control, showing likely the hybrid state of EMT.

Furthermore, at the protein level, the expression of Vimentin, Cadherin‐2, and Twist‐related protein 1 (H‐twist) was increased in COMP‐BT20 (Fig. 1C,D) and COMP‐HS578T cells (Fig. S1A) compared with the mock counterparts. Cadherin‐1 expression was also reduced in COMP‐BT20 (Fig. 1C,D), but it was undetectable in HS‐578 T cells (Fig. S1A).

Moreover, paraffin‐embedded tissues derived from primary tumors of our previous xenograft mouse model [16] showed increased expression of Vimentin and Matrix metalloproteinase‐9 (MMP9) in tumors expressing COMP in comparison with the mock control tumors (Fig. 1E with quantification in Fig. 1F). In brief, the xenograft mouse model was performed by injecting COMP‐expressing MDA‐MB‐231 cells or the mock control cells into SCID mice orthotopically. A pool of four stable clones for COMP‐expressing cells or the mock control, with an equal proportion of each clone, was injected into mice. COMP‐mice showed significantly larger tumors along with metastases to lungs and lymph nodes compared with the mock control mice [16]. The protein markers, SNAI2 and Cadherin‐1, were also examined without revealing any statistically significant differences between COMP or mock tumor tissues (Fig. S1B). Taken together, COMP overexpression was found to contribute to the mesenchymal state of breast cancer cells.

### 3.2 COMP induces EMT in both autocrine and paracrine manners

**Fig. 2:**
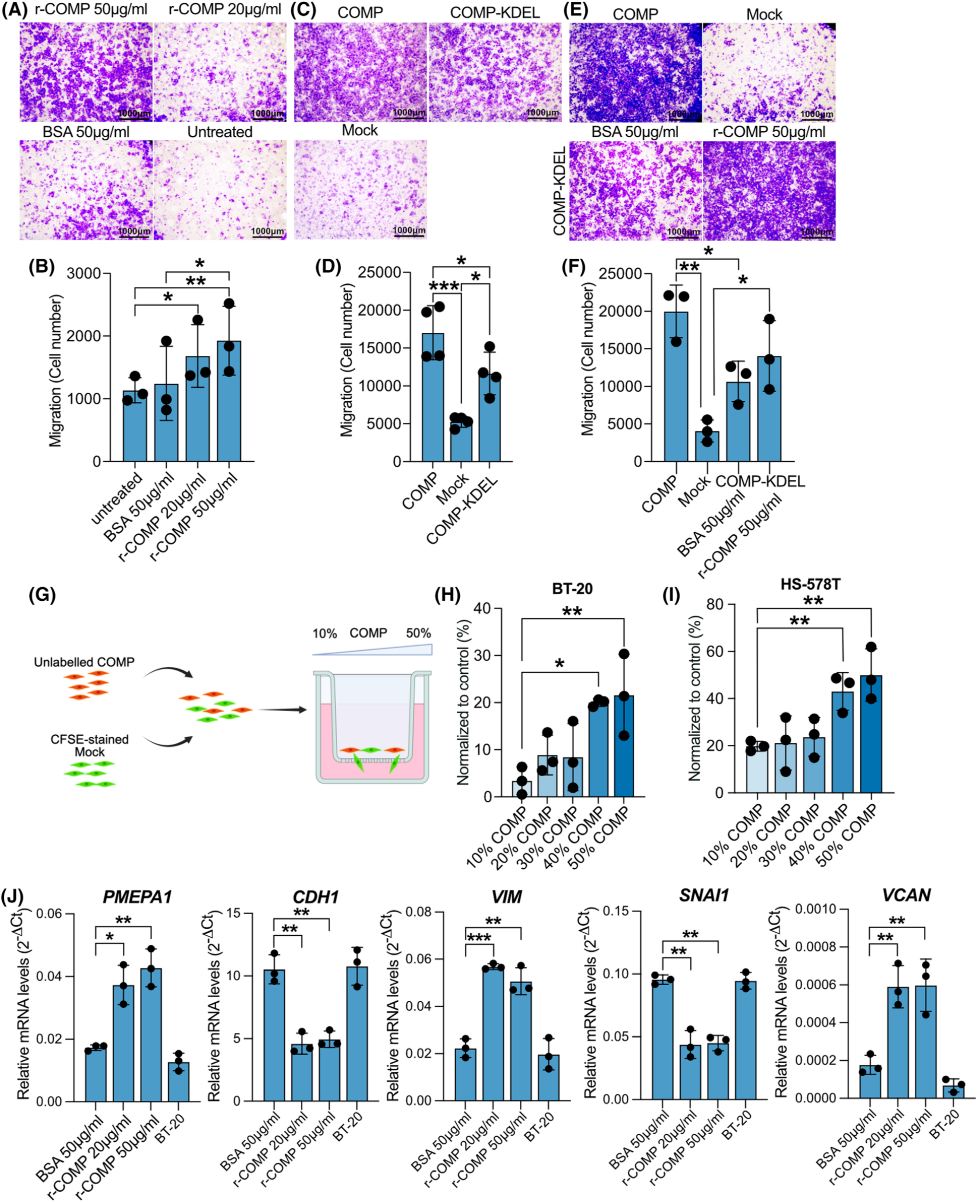
COMP‐induced EMT in both autocrine and paracrine manners. (A) A representative migration assay using BT‐20 cells treated with r‐COMP (20 or 50 μg·mL^−1^) and BSA (50 μg·mL^−1^) as a control for 48 h (scale bar: 1000 μm), quantified in (B). Data represent mean ± standard deviation from three independent experiments (*n* = 3). (C) A representative migration assay using COMP‐KDEL‐BT20, COMP‐BT20, and the mock control cells (scale bar: 1000 μm), quantified in (D). Data represent mean ± standard deviation from four independent experiments (*n* = 4). (E) A representative migration assay using COMP‐KDEL‐BT20 cells treated with r‐COMP (50 μg·mL^−1^) or BSA (50 μg·mL^−1^), COMP‐BT20, and the mock control cells (scale bar: 1000 μm), quantified in (F). Data represent mean ± standard deviation from three independent experiments (*n* = 3). (G) A representative scheme for the cocultured migration assay using CFSE‐labeled mock cells and the COMP‐expressing cells. Migrated CFSE‐labeled mock cells were captured using fluorescent microscope and quantified with QuPath software. The statistical analyses for the migrated CFSE‐labeled mock cells cocultured with COMP‐BT20 (H) and COMP‐HS578T (I). Data represent mean ± standard deviation from three independent experiments (*n* = 3). (J) The expression analyses of EMT markers by RT‐qPCR in BT‐20 cells treated with r‐COMP (20 or 50 μg·mL^−1^), BSA (50 μg·mL^−1^), and the untreated cells. *HPRT* was used as a reference gene. Data represent mean ± standard deviation from three independent experiments (*n* = 3). *P*‐values were calculated using one‐way ANOVA for all graphs. *P ≤ 0.05, **P ≤ 0.01, and ***P ≤ 0.001. Non‐significant differences were not shown. EMT: Epithelial‐to‐mesenchymal transition, COMP‐KDEL cells: Cells that retain COMP in the endoplasmic reticulum.

To functionally dissect the role of COMP in EMT induction, a migration assay supplementing the culture medium with r‐COMP (20 and 50 μg·mL^−1^) or BSA as a control (Fig. 2A) was performed. R‐COMP significantly increased the number of migrated BT‐20 cells compared with untreated cells (Fig. 2B). A similar observation was made for MCF‐7 cells expressing COMP compared to mock controls (Fig. S1J).

Although COMP is a secreted protein, it plays a significant role in resistance to chemotherapy when present intracellularly, as demonstrated using the COMP‐KDEL‐expressing BT‐20 cells. These cells retain COMP in the ER and were characterized previously [32]. They express a form of COMP protein, in which the KDEL peptide was inserted before the stop codon, ensuring that the protein remains within the ER. COMP‐KDEL cells had significantly higher migration capability than the mock control but lower than COMP‐BT20 cells (Fig. 2C,D). The migration assay was then performed using the COMP‐KDEL cells cultured in the presence of r‐COMP (Fig. 2E). The migration of these cells was not significantly lower than that of COMP‐expressing cells (*P* = 0.204) (Fig. 2F). Thus, both intracellular and extracellular COMP contribute to the induction of migration. To investigate whether COMP exerts a paracrine effect, mock BT‐20 and mock HS‐578 T cells were labeled with CFSE and mixed with COMP‐BT20 and COMP‐HS‐578 T, respectively, at varying proportions of COMP‐expressing cells, ranging from 10 to 50%, and used for a migration assay (Fig. 2G). Results indicated that the higher the ratio of COMP‐BT20 (Fig. 2H) or COMP‐HS578T (Fig. 2I) cells, the higher the number of migrated cells. In addition, r‐COMP treatment (20 and 50 μg·mL^−1^) of BT‐20 cells, similar to the endogenous COMP expression (Fig. 1B), influenced the expression of EMT markers in favor of a reduced epithelial status; *CDH1* downregulation, and an increased mesenchymal status; *VIM* and *VCAN* upregulation. While *SNAI1* was also downregulated (Fig. 2J).

### 3.3 EMT‐related genes are upregulated in COMP‐expressing breast cancer cell lines

**Fig. 3:**
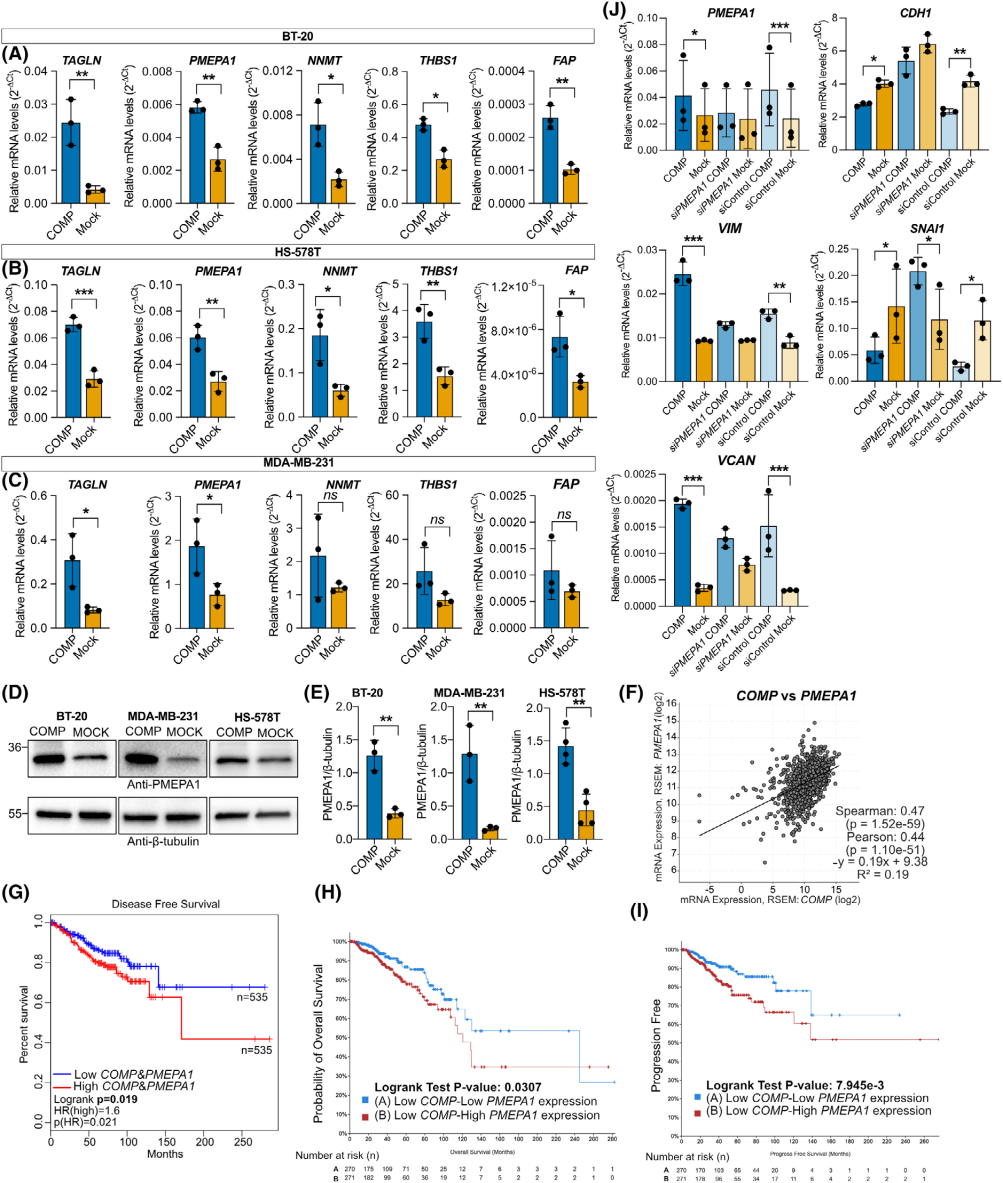
*PMEPA1* expression was positively correlated with *COMP* expression in breast cancer cell lines and patient cohorts. RT‐qPCR gene expression analyses of five genes involved in the EMT pathway, in three different breast cancer cell lines (A) BT‐20, (B) HS‐578 T, and (C) MDA‐MB‐231. *HPRT* was used as a reference gene. *P*‐values were calculated using a *t*‐test. Data represent mean ± standard deviation from three independent experiments (*n* = 3). (D) Representative western blots for protein TMEPA1 expression in breast cancer cell lines, quantified in (E). *P*‐values were calculated using a t‐test. Data represent mean ± standard deviation from at least three independent experiments. (F) *COMP*‐*PMEPA1* co‐expression in breast cancer patients, retrieved from an mRNA sequencing data using the cBioPortal. (G) Disease‐free survival analysis in breast cancer patients, stratified into two groups, low *COMP* and *PMEPA1* expression (dual low) versus high *COMP* and *PMEPA1* expression (dual high) in tumor tissues, assessed by the GEPIA2 online tool. (H, I) The dependency of the prognostic effect was then evaluated. Patients were initially stratified into two groups of low *COMP* and high *COMP* expression, then in each group, patients were divided into low *PMEPA1* and high *PMEPA1* expression. The probability of overall survival (H) and progression‐free survival (I) analyses using the cBioPortal, in breast cancer patients stratified initially by the level of *COMP* expression and had low *COMP* expression, and secondly stratified by *PMEPA1* expression, either low or high *PMEPA1*. (J) The expression analyses of EMT markers by RT‐qPCR in COMP‐BT20 and the mock control, along with their respective counterparts treated with *PMEPA1*‐targeting siRNA (*siPMEPA1*) or scrambled control (siControl). *HPRT* was used as a reference gene. Data represent mean ± standard deviation from three independent experiments (*n* = 3). *P*‐values were calculated using One‐Way ANOVA. *P ≤ 0.05, **P ≤ 0.01, and ***P ≤ 0.001. EMT: Epithelial‐to‐mesenchymal transition.

Based on the EMT pathway altered genes, identified in our previous microarray analysis of COMP‐expressing tumors [16] and their association with COMP, five genes were selected for further evaluation: *TAGLN* (Transgelin), *PMEPA1*, *NNMT* (Nicotinamide N‐Methyltransferase), *THBS1* (Thrombospondin 1), and *FAP* (Fibroblast Activation Protein Alpha). The transcriptional activation of these genes was examined by RT‐qPCR in breast cancer cell lines, BT‐20, MDA‐MB‐231, and HS‐578 T, aiming to verify the observed differences. All five genes were significantly upregulated in COMP‐BT20 and COMP‐HS578T cells compared with the mock counterparts (Fig. 3A,B). In COMP‐MDA‐MB‐231 cells, the increased expression of only *TAGLN* and *PMEPA1* was statistically significant, while a similar tendency for *NNMT*, *THBS1*, and *FAP* was observed (Fig. 3C).

### 3.4 *PMEPA1* expression is positively correlated with *COMP* expression in breast cancer cell lines and patient cohorts

Among the five validated genes in the EMT pathway, *PMEPA1* was selected for further investigation due to its association with TGFβ signaling pathway [26], and the known COMP–TGFβ feedback loop [33, 34]. In all three cell lines, PMEPA1 was significantly elevated in COMP‐expressing cells compared to their mock counterparts (Fig. 3D, E). In addition, analysis of breast cancer datasets in cBioPortal [29] revealed a positive correlation between *COMP* and *PMEPA1* expression (Fig. 3F). A survival analysis via the GEPIA2 online tool [30] indicated that patients with tumors that expressed high levels of *COMP* and *PMEPA1* simultaneously (dual high) had significantly shorter disease‐free survival compared to patients with low levels of *COMP* and *PMEPA1* expression (dual low) (*P* = 0.019, Fig. 3G). Then, to address the dependency of this prognostic effect on individual *COMP* or *PMEPA1* expression, patients were stratified according to each gene expression (median) individually, and the mixed states were examined using cBioPortal. Significantly shorter overall survival and progression‐free survival were observed only in patients with tumors characterized by low *COMP* expression, which exhibited high *PMEPA1* expression (Fig. 3H,I, Fig. S1D–I), indicating that the prognostic relevance of *PMEPA1* is dependent on *COMP* expression levels, as in patients with high *COMP* expression, the prognostic effect of *PMEPA1* was lost.

Thereafter, the involvement of *PMEPA1* in the regulation of EMT markers was investigated in BT‐20 cells. The reduction of *PMEPA1* expression using siRNA (*siPMEPA1*) abrogated the significant differences between the COMP–BT20 cells and the mock control cells for the assessed EMT markers, *CDH1*, *VIM*, and *VCAN* and significantly reversed for *SNAI1* (Fig. 3J). These results suggest that *PMEPA1* expression in breast cancer cell lines and tumor tissues was positively correlated with *COMP* and that *PMEPA1* expression is necessary for the expression of *COMP*‐induced EMT markers.

### 3.5 *PMEPA1* downregulation abrogates the pro‐migratory and pro‐invasive effect of COMP on breast cancer cells

**Fig. 4:**
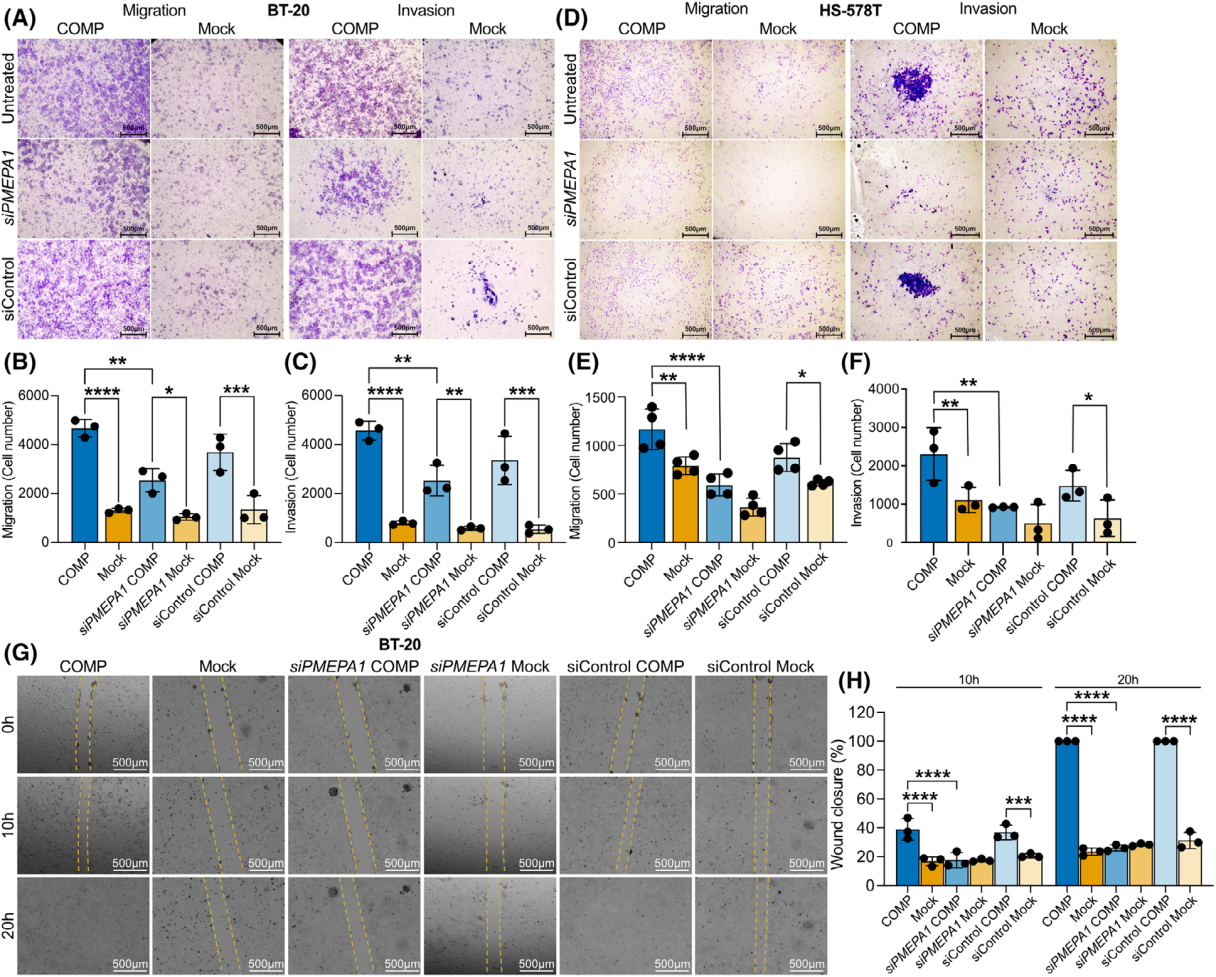
*PMEPA1* downregulation impaired the migration and invasion ability of cells induced by COMP. (A) Representative migration and invasion assays using COMP‐BT20 and the mock control, along with their respective counterparts treated with *siPMEPA1* or siControl (scale bar: 500 μm), and the statistical analyses; the migration assay (*n* = 3) (B) and the invasion assay (*n* = 3) (C). Similar assays (scale bar: 500 μm) (D) and statistical analyses for HS‐578 T cells; the migration assay (*n* = 4) (E) and the invasion assay (*n* = 3) (F). One‐Way ANOVA was used for the statistical analyses. Data represent mean ± standard deviation from at least three independent experiments. (G) A representative wound healing assay using COMP‐BT20 and the mock control, along with their respective counterparts treated with *siPMEPA1* or siControl (scale bar: 500 μm), quantified in (H). The wound closure (%) was calculated by the ImageJ software. Two‐way ANOVA was utilized for the statistical analysis. Data represent mean ± standard deviation from three independent experiments (*n* = 3). *P ≤ 0.05, **P ≤ 0.01, ***P ≤ 0.001 and ****P ≤ 0.0001. Non‐significant differences were not shown.

The regulatory role of PMEPA1 in COMP‐induced EMT was functionally assessed by siRNA downregulation of *PMEPA1* in COMP‐expressing cells, followed by migration, invasion, and wound healing assays. Efficiency of knockdown was assessed using RT‐qPCR and western blotting (Fig. S1K). When *PMEPA1* was downregulated in COMP‐expressing cells, the number of migrated BT‐20 cells (Fig. 4A,B) and HS‐578 T cells (Fig. 4D,E) was significantly reduced compared with the COMP‐expressing cells. In an invasion assay, COMP‐BT20 (Fig. 4A,C) and COMP‐HS578T (Fig. 4D,F) cells exhibited significantly increased invasion through Matrigel in comparison with the mock counterparts. Like the migration assay, a significantly reduced number of invaded *siPMEPA1*‐COMP BT‐20 (Fig. 4C) and *siPMEPA1*‐HS578T (Fig. 4F) cells were observed compared with their COMP‐expressing counterparts. Similarly, a wound healing assay indicated that COMP‐BT20 (Fig. 4G,H) and COMP‐HS578T cells (Fig. S1C) migrated significantly faster than the mock control, and *PMPEA1* downregulation in COMP‐BT20 cells abolished the observed difference between COMP‐BT20 and the mock control cells.

### 3.6 The C‐terminal region and the TSP domains of COMP protein are critical for its ability to induce EMT

**Fig. 5:**
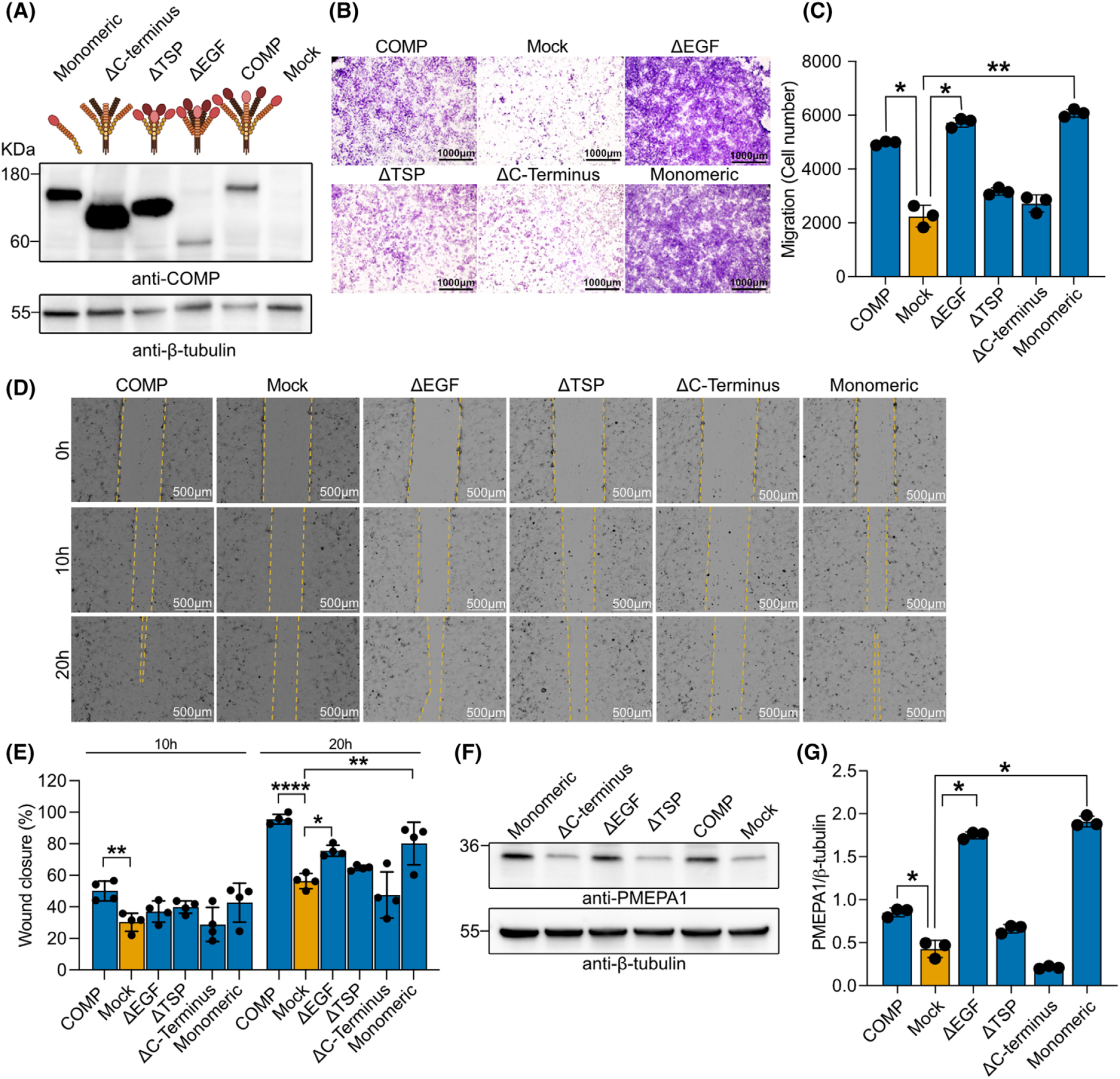
COMP mutants‐expressing BT‐20 cells showed different levels of migration ability and PMEPA1 expression. (A) A representative western blot visualizing the deletion mutants of COMP stably expressed in BT‐20 cells. These mutants were characterized in our previous study [32] (B) A representative migration assay using the cells expressing COMP mutants (scale bar: 1000 μm), quantified in (C) and analyzed using one‐way ANOVA. Data represent mean ± standard deviation from three independent experiments (*n* = 3). (D) A wound healing assay using the same COMP mutant‐expressing cells (scale bar: 500 μm), quantified in (E) and analyzed using two‐way ANOVA. Data represent mean ± standard deviation from four independent experiments (*n* = 4). Contrast was adjusted identically for all images in panel D; differences in image brightness reflect acquisition‐related variation generated by the Cytation system (e.g., well position) and were not adjusted. (F) A representative western blot for PMEPA1 expression in COMP mutant‐expressing cells, quantified in (G) and analyzed using one‐way ANOVA. Data represent mean ± standard deviation from three independent experiments (*n* = 3). *P ≤ 0.05, **P ≤ 0.01, and ****P ≤ 0.0001. Non‐significant differences were not shown. Monomeric, ΔTSP, ΔEGF, and ΔC‐Terminus: BT‐20 cells stably expressing deletion mutants of COMP in these regions including the N‐terminal polymerization region (monomeric), the TSP domains (ΔTSP), the EGF repeats (ΔEGF), and the C‐terminal globular region (ΔC terminus). TSP: Thrombospondin, EGF: epidermal growth factor.

To understand which COMP domains are responsible for its function, deletion mutants of *COMP* in these regions were constructed, including the N‐terminal polymerization region (monomeric), the TSP domains (ΔTSP), the EGF repeats (ΔEGF), and the C‐terminal globular region (ΔC‐Terminus). These mutants were expressed in BT‐20 cells (Fig. 5A) and characterized in our previous study [32]. The deletion of the C‐terminal region and the TSP domains reverted the pro‐migratory effect of COMP in BT‐20 cells (Fig. 5B,C). Similarly, a wound healing assay indicated that the same mutants abrogated the wound closure induction compared to the full‐length pentameric COMP (COMP‐BT20; Fig. 5D,E). Interestingly, PMEPA1 expression was not elevated in cells expressing ΔC‐Terminus and ΔTSP mutants compared with the mock control. In contrast, BT‐20 cells expressing the ΔEGF mutant or the monomeric form showed a two‐fold increase in PMEPA1 expression in comparison to COMP‐BT20 cells (Fig. 5F,G).

### 3.7 COMP binds to PMEPA1 through the ΔTSP domain

**Fig. 6:**
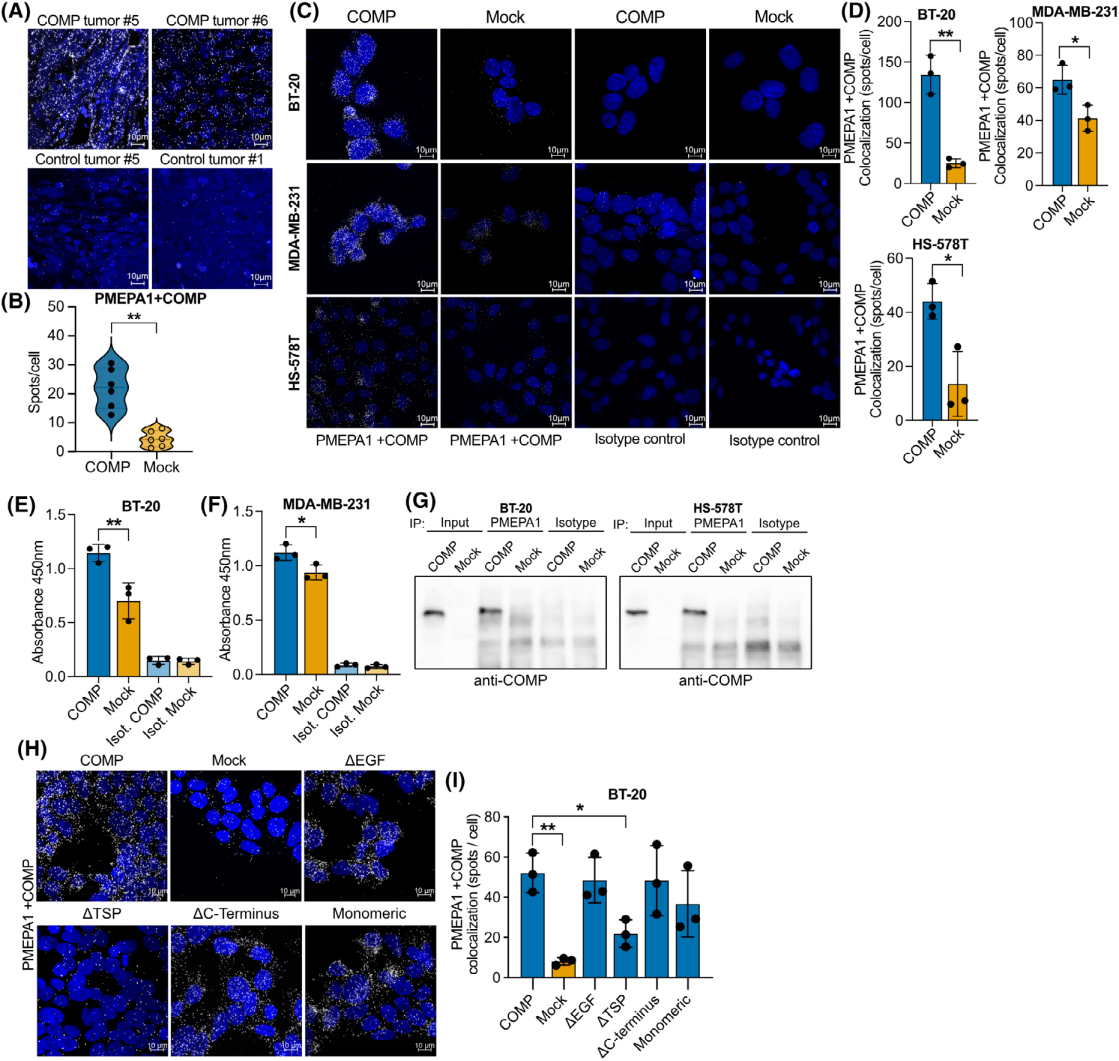
COMP binds PMEPA1 through its TSP domains. (A) A representative PLA for the COMP‐PMEPA1 interaction on paraffin‐embedded breast cancer tissues. Two images of COMP‐expressing tumors (from two different mice) and the control tumors (from two different mice) were represented (scale bar: 10 μm). (B) The spots/cell values were calculated by the ImageJ software, and the Mann–Whitney test was used for the statistical analysis. Each symbol in the graph represents a tumor from an individual mouse (*n* = 6). (C) PLA for COMP‐PMEPA1 interaction in three breast cancer cell lines, *in vitro* (scale bar: 10 μm), quantified in (D) and analyzed using t‐test. Data represent mean ± standard deviation from three independent experiments. Detection of the COMP‐PMEPA1 interaction by sandwich ELISA in (E) BT‐20 (*n* = 3) and (F) MDA‐MB‐231 cells (*n* = 3), analyzed using one‐way ANOVA. Data represent mean ± standard deviation from three independent experiments. (G) Co‐immunoprecipitation assay using BT‐20 and HS‐578 T cells confirming the COMP‐PMEPA1 interaction. (H) PLA for COMP‐PMEPA1 interaction in COMP mutant‐expressing cells (scale bar: 10 μm), quantified in (I) and analyzed using one‐way ANOVA. Data represent mean ± standard deviation from three independent experiments (*n* = 3). *P ≤ 0.05, and **P ≤ 0.01. Non‐significant differences were not shown. Monomeric, ΔTSP, ΔEGF, and ΔC‐Terminus: BT‐20 cells stably expressing deletion mutants of COMP in these regions including the N‐terminal polymerization region (monomeric), the TSP domains (ΔTSP), the EGF repeats (ΔEGF), and the C‐terminal globular region (ΔC terminus). TSP: Thrombospondin, EGF: epidermal growth factor, PLA: Proximity ligation assay.

PMEPA1 is a transmembrane protein [35], while COMP is a secreted protein that subsequently rebinds to the cell membrane. To investigate whether the observed PMEPA1 regulatory role on COMP is due to a direct interaction between the two proteins, a proximity ligation assay (PLA) was performed in breast cancer tumors derived from a xenograft mouse model of our previous study [16]. A significantly higher number of dots per cell in COMP‐expressing tumor tissues was detected compared with the mock control tumors (Fig. 6A,B). PLA in COMP‐BT20, COMP‐MDA‐MB‐231, and COMP‐HS578T cells confirmed a significant interaction between COMP and PMEPA1 compared with the mock counterparts (Fig. 6C,D). Sandwich ELISA using BT‐20 (Fig. 6E) and MDA‐MB‐231 cells (Fig. 6F) and co‐immunoprecipitation assays using BT‐20 and HS‐578 T cells (Fig. 6G) further confirmed the interaction between COMP and PMEPA1 compared with the mock control cells and the isotype control. The specific band for COMP in the lysates from COMP‐expressing cells, co‐immunoprecipitated with PMEPA1 antibody, represents COMP‐ PMEPA1 interaction. In addition, the interaction between COMP and PMEPA1, assessed by PLA, was significantly reduced only in the ΔTSP mutant‐expressing BT‐20, indicating that this domain binds PMEPA1 (Fig. 6H,I).

### 3.8 COMP overexpression switches the TGFβ signaling pathway from phosphorylated SMAD2/3 (pSAMD2/3) to pSMAD1/5

**Fig. 7:**
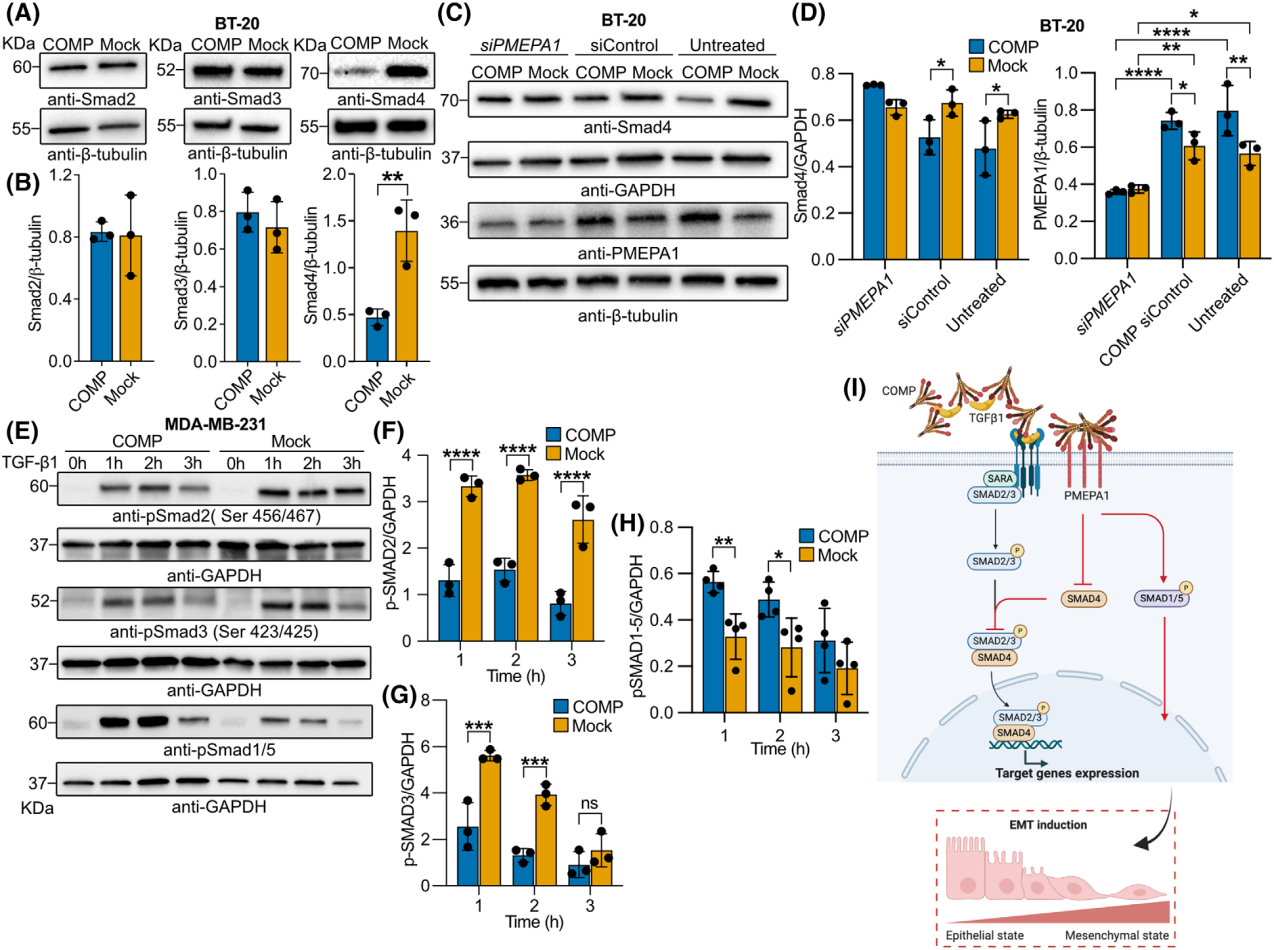
COMP reprogrammed the TGFβ signaling pathway toward pSMAD1/5 activity. (A) Representative western blots of total SMAD2, SMAD3, and SMAD4 expression in COMP‐BT20 versus the mock control cells, quantified in (B). *P*‐values were calculated using a t‐test. Data represent mean ± standard deviation from three independent experiments (*n* = 3). (C) A representative western blot for SMAD4 expression in the same cells, along with their respective counterparts treated with *siPMEPA1* or siControl, quantified in (D), which also shows efficiency of knockdown of *PMEPA1* on mRNA level. *P*‐values were calculated using a two‐way ANOVA. Data represent mean ± standard deviation from three independent experiments (*n* = 3). (E) Representative western blots showing the expression of pSMAD2 (Ser 456/467), pSMAD3 (Ser 423/425), and pSMAD1/5 (Ser 463/465) in COMP‐MDA‐MB‐231 and the mock control, treated with TGFβ1 (10 ng·mL^−1^) for 3 h, and the statistical analyses: pSMAD2 (*n* = 3) (F), pSMAD3 (*n* = 3) (G), and pSMAD1/5 (*n* = 4) (H). *P*‐values were calculated using a two‐way ANOVA. Data represent mean ± standard deviation from at least three independent experiments. (I) Schematic representation of the proposed mechanism created using Biorender. *P ≤ 0.05, **P ≤ 0.01, ***P ≤ 0.001 and ****P ≤ 0.0001. Non‐significant differences were not shown.

PMEPA1 interaction with COMP prompted the hypothesis that the expression of SMAD proteins, transcription factors in the TGFβ pathway, could be altered by COMP overexpression. The expression of total SMAD2 and SMAD3 remained unchanged in COMP‐BT20 cells compared with the mock control, while SMAD4 expression was significantly reduced (Fig. 7A,B). The observed reduction in SMAD4 expression was restored when PMEPA1 expression was downregulated, indicating the involvement of PMEPA1 (Fig. 7C,D). We then investigated whether the interaction between COMP and PMEPA1 affects the activation of receptor‐regulated SMADs, including SMAD2, SMAD3, and SMAD1/5 (Fig. 7E). COMP‐MDA‐MB‐231 cells showed lower levels of pSMAD2 (Ser 456/467; Fig. 7F) and pSMAD3 (Ser 423/425; Fig. 7G) expression compared with the mock control cells, stimulated with TGFβ1 (10 ng·mL^−1^) at all examined time points. Interestingly, the opposite trend was observed for the pSMAD1/5 expression (Fig. 7H). These results suggest that COMP overexpression shifts the canonical TGFβ pathway toward activation of pSMAD1/5 rather than pSMAD2/3, likely regulated by PMEPA1 overexpression. The proposed mechanism is illustrated in Fig. 7I.

## 4 Discussion

Our findings suggest the COMP–PMEPA1 axis as an EMT driver in breast cancer cells. COMP expression was positively associated with the expression of mesenchymal markers and the induction of migration and invasion abilities in breast cancer cells, acting in both autocrine and paracrine manners and dependent on TSP domains and the C‐terminal region of COMP. We identified a new functional COMP‐binding partner, PMEPA1, involved in mediating COMP's effect on EMT through binding to the TSP domains of COMP. In addition, COMP expression triggers a shift in the activity of the canonical TGFβ signaling pathway, specifically from a pSMAD2–pSMAD3‐dependent pathway to a pSMAD1/5‐dependent pathway in the presence of TGFβ.

Tumor microenvironment is a highly complex milieu with various alterations favoring tumor cells' progression and aggressiveness. Acquiring EMT signature, reduced epithelial and increased mesenchymal characteristics, is accompanied by multiple other changes such as aberrant gene expression, ECM remodeling, altered angiogenesis, and modification of tumor secretome [36]. Throughout the EMT continuum, including full epithelial, early EMT (partial EMT or hybrid state), and late EMT (full mesenchymal) stages, cells undergo dynamic changes in gene and protein expression, which may also vary depending on tissue context. According to the lineage tracing transgenic mouse models of breast cancer, attributing a specific marker to a particular stage of EMT, mostly to the mesenchymal stages, is not well‐defined and widely accepted, as different *in vivo* models may have their own limitations and experimental conditions. Although cadherin‐1 is an accepted epithelial marker, the activation of mesenchymal markers, such as Tenascin‐C, cadherin‐2, Vimentin, and Fibroblast‐specific protein 1 (FSP1), at early or late EMT, and their requirements in tumor growth and metastasis are still a matter of debate [37, 38, 39, 40]. For instance, cadherin‐2 has been expressed in primary tumors and most of the lung metastases in an N‐cad‐EMTracer‐MMTV‐PyMT mouse model of metastatic breast cancer, and its knockout significantly reduced the lung metastases [37]. In contrast, in another stud*y*, cadherin‐2, considered as a marker for full EMT, was found not to be associated with metastasis, but instead contributed to chemoresistance [38]. This study, therefore, covers a panel of EMT markers at the transcriptional and translational levels, supporting a comprehensive overview of EMT‐known markers and their alterations in breast cancer cell lines. We mainly focused on triple‐negative breast cancer (TNBC). BT‐20, HS 578 T, and MDA‐MB‐231 were selected as they represent complementary epithelial‐like and mesenchymal TNBC phenotypes, providing a broad EMT‐relevant spectrum.

Our data show a heterogeneous regulation of mesenchymal markers, with some (*FZD7*, *SNAI1*, *PTP4A1*, *CAMK2N1*) being downregulated while others are upregulated in COMP‐expressing cells. Such non‐uniform changes are characteristic of partial or hybrid EMT states, which are now recognized as biologically stable intermediates between epithelial and fully mesenchymal phenotypes [4]. Partial EMT frequently displays selective regulation of EMT‐associated genes rather than a coordinated mesenchymal program. Jagged‐dominated Notch pathway has been previously recognized as a crucial inducer of hybrid state in MDA‐MB‐231 cells by employing CD24‐CD44 double‐positive cells as a hybrid phenotype [41]. With regard to this, the association between hybrid EMT and breast cancer stemness has also been recognized [42]. Given our previously detected mechanism of COMP, i.e., Notch3 activation in COMP‐expressing breast cancer cells, MDA‐MB‐231 and BT‐20, COMP binding to both Notch3 and Jagged1, and COMP‐mediated cancer stem cells' induction [43], the observed heterogeneous regulation of mesenchymal markers could be attributed to the induction of hybrid state, not fully mesenchymal EMT by COMP. However, distinguishing between the hybrid state or the full mesenchymal EMT is beyond the scope of this study.

COMP is a well‐recognized oncoprotein in several types of solid cancers, such as breast [16], ovarian [21], and prostate cancers [17]. In breast cancer, high COMP expression in tumor cells, but not in the stroma, was associated with shorter overall and recurrence‐free survival, as determined by immunohistochemical analysis [16]. In addition, in a cohort of metastatic breast cancer patients, high COMP expression in lymph node metastases was associated with reduced overall survival [44]. COMP was found to protect breast cancer cells against apoptosis induced by endoplasmic reticulum (ER) stress, and to increase MMP9 expression, the invasive ability, and the Warburg effect [16]. A piece of recent evidence further highlights COMP's influence on EMT. Exosomes derived from type‐2 diabetic patients' adipocytes were found to induce EMT in breast cancer cell lines, showing an increased expression of mesenchymal markers such as *SNAI1*, *TWIST1*, *SNAI2*, *VIM*, *ZEB1*, and *TGFΒ1*, higher cell elongation and reduced circularity, which represents the mesenchymal cell phenotype, and a higher migration ability. Surprisingly, COMP was the most enriched protein in these exosomes, assessed by proteomics analysis [45]. The pro‐migratory and pro‐invasive role of r‐COMP, as well as its inhibitory and stimulatory impact on cadherin‐1 and Vimentin expression, respectively, has also been reported in HCC cell lines, where it was attributed to CD36 expression [23]. In the current study, we provided further confirmation of COMP involvement, both endogenously expressed and extracellularly added, in EMT induction, represented by the increased expression of several mesenchymal markers and higher migratory and invasion ability of breast cancer cells, particularly in TNBC cells. Moreover, the inclusion of MCF7 cells, an ER^+^/PR^+^ and low‐invasive cell line, further strengthened the role of COMP in EMT induction.

The extracellular matrix is increasingly recognized as an active regulator of cancer progression rather than a passive scaffold. Changes in ECM composition, stiffness, and organization can directly influence EMT, cell motility, and metastatic dissemination [46]. Matrix metalloproteinases are key mediators of ECM degradation and remodeling and have been implicated in both EMT induction and the acquisition of invasive traits in breast cancer. By proteolytic degradation of the basement membrane, MMPs trigger the disruption of cell‐ECM interaction and lead to the migration and invasion enhancement of tumor cells [36]. In our study, two MMPs, MMP9 and *MMP3*, were increased in COMP‐tumor tissues and COMP‐expressing cancer cell line, respectively. As a matrix protein, COMP is well positioned to participate in this crosstalk between ECM architecture and EMT programs. The COMP‐dependent changes that we observe in EMT‐related genes and invasive properties suggest that COMP may contribute to a pro‐invasive ECM niche that supports partial or full EMT. Modulation of COMP affected the expression/activities of MMPs, further linking COMP to ECM remodeling and EMT‐related functions. Together, these data support a model in which COMP promotes breast cancer aggressiveness by coordinating ECM structure with EMT‐associated transcriptional and functional changes.

In addition, for the first time, we have shown that the TSP domains and C‐terminal region of COMP are responsible for these activities. The TSP domains are also essential for COMP‐induced chemoresistance in breast cancer cell lines in which COMP expression confers resistance to multiple chemotherapeutic agents, anti‐HER2 therapy, and endocrine treatment, regardless of the breast cancer subtypes, primarily through its localization in the endoplasmic reticulum and interaction with calpain [32]. Given the growing evidence linking EMT to chemoresistance [47] and the established role of COMP in promoting chemoresistance in breast cancer cells [32], the EMT induction by COMP observed in this study appears well‐founded.

The five genes (*NNMT*, *TAGLN*, *THBS1*, *FAP*, *PMEPA1*) identified by the microarray analysis as altered in COMP‐expressing tumors and the previous evidence linking each gene to *COMP* were further validated in COMP‐expressing breast cancer cell lines. *NNMT* has been shown to regulate COMP transcriptionally; both *NNMT* knockdown (*shNNMT*) and its overexpression positively modulate *COMP* expression in CAFs isolated from tumor‐containing omental tissue of ovarian cancer patients and normal fibroblasts [48]. COMP has also been reported to interact and colocalize with transgelin in colorectal cancer cell lines and tissues, a relationship associated with enhanced EMT in COMP‐expressing colorectal cancer cells. Chrysin, an inhibitor of the COMP‐transgelin complex, significantly reduced the expression of COMP and suppressed EMT in COMP‐expressing cells [49]. Thrombospondin‐1 (encoded by *THBS1*) was included because like COMP, it belongs to the thrombospondin family; however, it forms a trimeric structure rather than the pentameric structure characteristic of COMP [50]. Although fibroblast activation protein alpha (encoded by *FAP*) is highly expressed in fibroblasts, evidence shows its expression in epithelial cells as well, associated with invadopodia stabilization and tumor metastasis [51]. We found it interesting as fibroblast activation protein alpha is also involved in ECM remodeling and its expression was positively correlated with *COMP* and collagen type I alpha 1 chain (*COL1A1*) in breast tumor tissues analyzed by GEPIA2 (data not shown). Among these genes, *PMEPA1* was selected for further investigation. PMEPA1 is a target of the TGFβ signaling pathway, which becomes overexpressed in the presence of TGFβ but not bone morphogenetic protein (BMP). COMP interaction with TGFβ1 has also been revealed previously [52]. PMEPA1 can interact with SMAD2 and SMAD3 proteins and prevent their complex formation with SMAD4, leading to suppression of the TGFβ signaling pathway. This suppressive effect of PMEPA1 was due to the competition between PMEPA1 and the Smad anchor for receptor activation (encoded by *SARA*) for binding to the SMAD2 tryptophan^368^ residue [53]. The Smad anchor for receptor activation recruits SMADs to become phosphorylated by activated TGFβRI. Although SMAD4 does not directly interact with PMEPA1 [53], in MDA‐MB‐468 breast cancer cells lacking both PMEPA1 and SMAD4 in the presence of TGFβ, SMAD4 overexpression restored PMEPA1 expression, indicating that SMAD4 is required for PMEPA1 expression. In contrast, *PMEPA1* knockdown by shRNA in MDA‐MB‐231 and HCC1937 cells had no effect on SMAD4 levels [54]. Our results indicated that SMAD4 expression was significantly reduced in COMP‐BT20 and that downregulation was restored with siRNA against *PMEPA1*. Having demonstrated this effect, the observed PMEPA1 overexpression in COMP‐expressing breast cancer cells suggests that sustained PMEPA1 expression may suppress SMAD4, potentially in a cell context‐dependent manner. In addition to the SMAD4 suppression, pSMAD2 and pSMAD3 expression were also decreased in COMP‐expressing cells, while pSMAD1/5 was significantly increased, indicating that COMP overexpression reprograms the TGFβ signaling pathway, likely due to the PMEPA1 sustained overexpression, as this effect was consistent with previous findings showing PMEPA1 overexpression in HCT116 cells, a colon cell line, concomitant with reduced pSMAD2 and pSMAD3 expression with an increased expression in pSMAD1/5 in the presence of TGFβ [27]. Same changes in the expression of pSMAD2 and pSMAD3 upon *PMEPA1* downregulation or upregulation were observed in MDA‐MB‐231 and HCC1937 breast cancer cells and in HMEC cells, a primary mammary epithelial cell line [54].

Considering the SMAD4 regulatory role in the receptor‐regulated SMADs' function, and its suppression in COMP‐expressing cells, we cannot yet rule out the functional contribution of TGFβ signaling pathway via pSMAD1/5 in the observed COMP‐PMEPA1 mediated EMT, as it has been reported that other EMT‐transcription factors like Zinc finger protein SNAI1, in the presence of mutated SMAD4, can induce EMT in colorectal cancer cell lines independent of TGFβ or BMP signaling. This suggests that signaling pathways leading to EMT are subjected to genetic and epigenetic plasticity, highlighting the complexity of EMT regulation [55, 56]. Furthermore, non‐canonical SMAD‐independent TGFβ signaling pathways may become activated in the presence of sustained PMEPA1 expression in COMP‐expressing cells, which remained exclusive in the current study. Also, analyzing the breast cancer patient database revealed that *PMEPA1* prognostic relevance is context‐dependent, relying on *COMP* expression levels. Although patients with high expression of both *COMP* and *PMEPA1*, simultaneously, exhibited shorter disease‐free survival compared to those with low expression of both genes, the dependency analyses of the prognostic effect on COMP indicated that when COMP expression is high, the prognostic relevance of *PMEPA1* is not evident any longer. This suggests that elevated *COMP* expression may exert a dominant biological effect, potentially through COMP‐associated ECM remodeling [18] or COMP‐mediated signaling pathways like Notch3‐jagged1 [43], which could mask the *PMEPA1* effect. Consequently, *PMEPA1* prognostic effect was exclusively evident in tumors with low *COMP* expression. These findings underscore the importance of stratifying patients by *COMP* levels before assessing *PMEPA1*‐associated prognosis.

## 5 Conclusions

Taken together, this study identifies PMEPA1 as a downstream target in the COMP‐mediated signaling pathway and reveals its regulatory role in COMP‐induced EMT via binding to COMP's TSP domains.

## Conflict of interest

The authors declare no conflict of interest.

## Author contributions

KSP and AB were responsible for the study design. KSP, GGB, and AB wrote the manuscript. KSP analyzed data, interpreted results, and conducted the experiments. GGB performed database analyses. LG, JZ, KG, and YTN performed the experiments. AB has full access to the data in the study and acquired the funding.

## Supporting information


**Fig. S1.** Expression of EMT markers in HS‐578 T cells and tumor tissues, wound healing assay using COMP‐HS578T cells or the mock control, the dependency of the prognostic effect on individual *COMP* or *PMEPA1*, migration assay using COMP‐MCF7 cells or the mock control, and the efficiency of *PMEPA1* knockdown in BT‐20 cells.
**Table S1.** List of the used probes.
**Table S2.** List of the used antibodies.

## Data Availability

The data that support the findings of this study are available from the corresponding author [anna.blom@med.lu.se] upon reasonable request.
