# Supplementary material for: COMP–PMEPA1 axis promotes epithelial‐to‐mesenchymal transition in breast cancer cells
Source: Mol Oncol. 2026 Feb 13;20(7):1780–99. doi: 10.1002/1878-0261.70221 (PMC13352958; doi:10.1002/1878-0261.70221)
Supplement: Supplementary file 1 — Fig. S1. Expression of EMT markers in HS‐578 T cells and tumor tissues, wound healing assay using COMP‐HS578T cells or the mock control, the dependency of the prognostic effect on individual COMP or PMEPA1, migration assay using COMP‐MCF7 cells or the mock control, and the efficiency of PMEPA1 knockdown in BT‐20 cells. Table S1. List of the used probes. Table S2. List of the used antibodies. [file MOL2-20-1780-s001.pdf]

Supplementary information:

Supplementary Figure 1

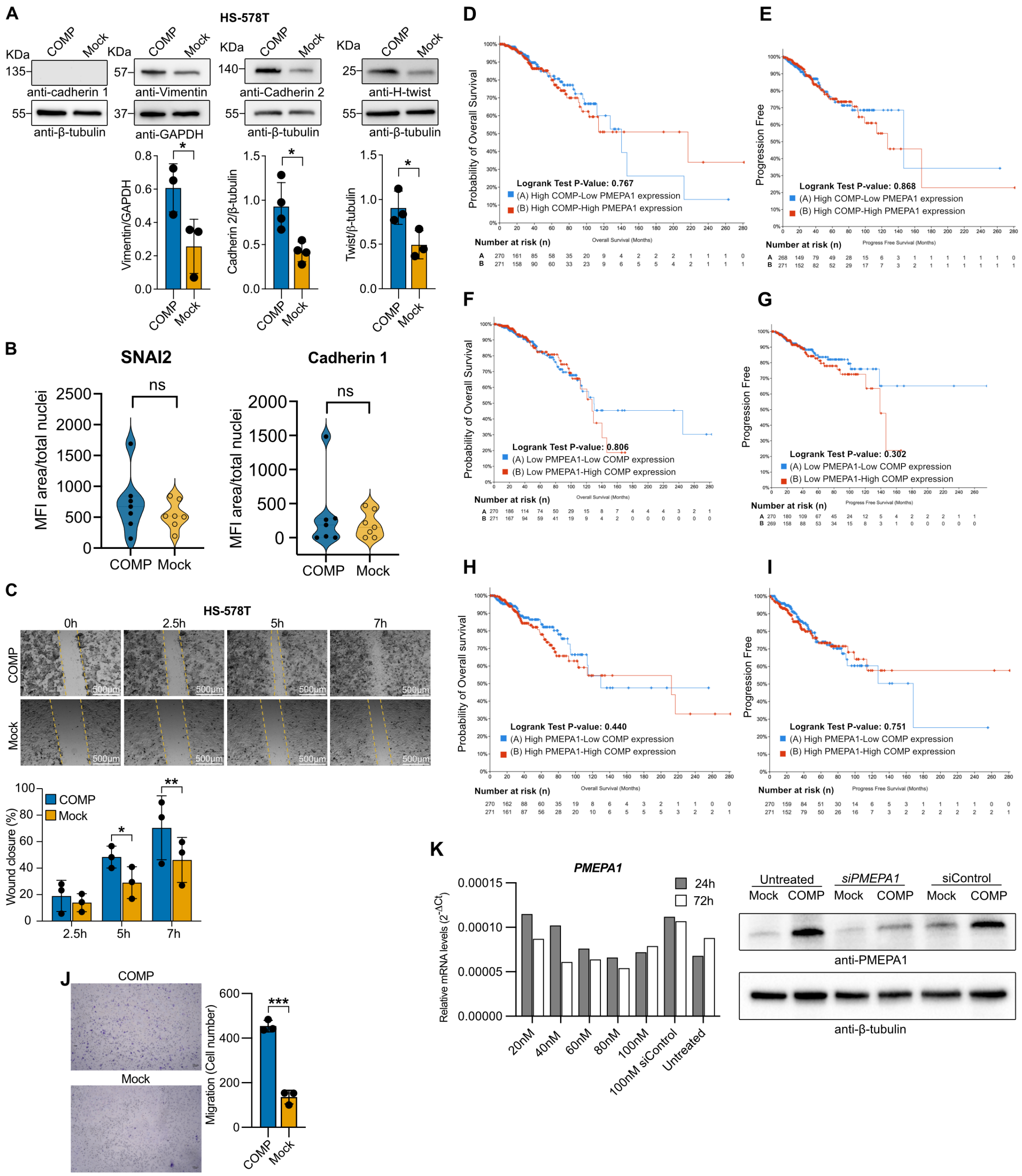

Supplementary Figure 1. Expression of EMT markers in HS-578T cells and tumor tissues, wound healing assay using COMP-HS578T cells or the mock control, the dependency of the prognostic effect on individual *COMP* or *PMEPA1*, migration assay using COMP-MCF7 cells or

the mock control, and the efficiency of *PMEPA1* knockdown in BT-20 cells. (**A**) Representative western blots showing the expression of EMT markers in COMP-HS578T or the mock control cells, and the statistical analyses. Cadherin-1 was not detected in HS-578T cells. Statistical analysis was performed using a t-test. Data represent mean±SD from at least three independent experiments. *$p \leq 0.05$ (**B**) Statistical analyses for Zinc finger protein SNAI2 and cadherin-1 immunofluorescence staining, in paraffin-embedded tumor tissues collected from a previously performed xenograft mouse model. Each symbol in the graph represents a tumor from an individual mouse (n=7). The Mann-Whitney test was used for the statistical analysis. ns: non-significant (**C**) A wound healing assay using COMP-HS578T or the mock control cells incubated for 7 hr, and the statistical analysis. *P*-values were calculated using a Two-way ANOVA. Data represent mean±SD from three independent experiments. *$p \leq 0.05$, and **$p \leq 0.01$. (**D, E**) The probability of overall survival and progression-free survival analyses in breast cancer patients using the cBioPortal. Patients were initially stratified into two groups of high *COMP* and low *COMP* expression, then in each group, patients were divided into low *PMEPA1* and high *PMEPA1* expression. No prognostic value for *PMEPA1*, either for the probability of overall survival or the progression-free survival, was detected when *COMP* was highly expressed. (**F-I**) Similar survival analyses while patients were initially stratified into low *PMEPA1* (**F, G**) and high *PMEPA1* (**H, I**) expression, then in each group, patients were divided into low *COMP* and high *COMP* expression as a second factor. *PMEPA1* showed a prognostic value only when *COMP* was not highly expressed. (**J**) Migration of MCF-7 cells expressing COMP vs mock controls. Statistical analysis was performed using a t-test. Data represent mean±SD from three independent experiments. ***$p \leq 0.001$. (**K**) Efficiency of knockdown of *PMPEA1* in BT-20 upon transfection with indicated concentrations of siRNA. mRNA was measured using RT-qPCR, while protein level was assessed by Western blotting, detection of β-tubulin served as the loading control.

Table S1. List of the used probes

Target	Supplier	Catalogue number
CDH1	Applied Biosystem	Hs01023895_m1
DSP	Applied Biosystem	Hs00950591_m1
SNAI1	Applied Biosystem	Hs00195591_m1
SNAI2	Applied Biosystem	Hs00161904_m1
FN1	Applied Biosystem	Hs01549976_m1
MMP3	Applied Biosystem	Hs00968305_m1
TGFB2	Applied Biosystem	Hs00234244_m1
VIM	Applied Biosystem	Hs00958111_m1
CDH2	Applied Biosystem	Hs00983056_m1
FZD7	Applied Biosystem	Hs00275833_s1
TWIST1	Applied Biosystem	Hs01675818_s1
SERPINE1	Applied Biosystem	Hs00167155_m1
VCAN	Applied Biosystem	Hs00171642_m1
KRT19	Applied Biosystem	Hs00761767_s1
PTP4A1	Applied Biosystem	Hs06633630_g1
CALD1	Applied Biosystem	Hs00921987_m1
CAMK2N1	Applied Biosystem	Hs00218591_m1
TAGLN	Applied Biosystem	Hs01038777_g1
PMEPA1	Applied Biosystem	Hs00375306_m1
NNMT	Applied Biosystem	Hs00196287_m1
THBS1	Applied Biosystem	Hs00962908_m1
FAP	Applied Biosystem	Hs00990791_m1

Table S2. List of the used antibodies

Target	Subtype	Supplier	Catalogue number
COMP	Polyclonal Rabbit IgG	Homemade	-
COMP	Polyclonal Goat IgG	R&D	AF3134
Rabbit IgG HRP	Polyclonal Goat IgG	Dako	P0448
Rabbit IgG HRP	Polyclonal Goat IgG	CST	7074
β-tubulin	Polyclonal Rabbit	Abcam	Ab6046
GAPDH	Monoclonal Mouse	Abcam	8245
Rabbit Isotype	Polyclonal Rabbit	Merck Millipore	12-370
Mouse IgG1, κ Isotype Ctrl	Monoclonal Mouse	Biolegend	400102
Vimentin	Monoclonal Mouse	Sigma Aldrich	V6389
protein TMEPAI	Monoclonal Mouse	Santa Cruz	SC-293372
protein TMEPAI	Polyclonal Rabbit	Abcam	Ab128006
Cadherin-1	Monoclonal Mouse	BD Transduction Laboratories	61082
Cadherin-2	Monoclonal Rabbit	CST	13116
H-twist	Monoclonal Mouse	Abcam	Ab50887
MMP9	Monoclonal Rabbit	Abcam	Ab76003
Alexa fluor 488 anti-rabbit	Donkey Polyclonal	Thermo Fisher Scientific	A32790
Alexa fluor 555 anti-mouse	Donkey Polyclonal	Thermo Fisher Scientific	A32773
Smad2	Monoclonal Rabbit	CST	5339
Smad3	Monoclonal Rabbit	CST	9523
Smad4	Monoclonal Rabbit	CST	38454
pSmad2 (Ser 456/467)	Monoclonal Rabbit	CST	3108
pSmad3 (Ser 423/425)	Monoclonal Rabbit	CST	9520
pSmad1/5	Monoclonal Rabbit	CST	13820
